# Identified needs in antimicrobial stewardship education for pediatric advanced practice providers: a qualitative analysis

**DOI:** 10.1017/ash.2025.10278

**Published:** 2026-01-20

**Authors:** Nadia Hill, Jade C. Riopelle, Molly Eron, Yasaman Fatemi, Kristin D. Maletsky

**Affiliations:** 1 Department of Pediatrics, Children’s Hospital of Philadelphia, Philadelphia, PA, USA; 2 School of Medicine, University of Limerick, Limerick, Ireland; 3 Perelman School of Medicine at the University of Pennsylvania, Philadelphia, PA, USA; 4 Department of Pediatrics, University of Washington School of Medicine, Seattle, WA, USA; 5 Seattle Children’s Research Institute, Seattle, WA, USA

## Abstract

**Introduction:**

There is limited research investigating advanced practice provider (APP) knowledge and perspectives on education in antimicrobial stewardship (AS).

**Setting:**

Large academic children’s hospital.

**Participants:**

APPs in Division of Pediatrics, Antimicrobial Stewardship Program (ASP) stakeholders.

**Objective design:**

We conducted four focus groups with APPs and one focus group with ASP stakeholders. APPs were asked eleven open questions about training, influences on prescribing practices, knowledge gaps and desired topics for education in AS as well as general barriers to learning. ASP stakeholders were asked five open-ended questions about teaching initiatives, knowledge gaps and high yield teaching topics for APPs.

**Results:**

20 APPs and 6 ASP stakeholders (1 medical director, 3 pharmacists and 2 pediatric infectious diseases fellows) participated in focus groups. Four domains and eight themes were generated. (1) Barriers to AS in Practice: lack of critical thinking and conflict between the ASP and APPs within the cultural context of the institution. (2) Approach to Education: logistical challenges to curriculum development and adopting APP centered approaches to teaching. (3) Education for New to Practice APPs: learning basics of microbiology, infectious diseases and utilizing resources to encourage AS in practice. (4) Education for Experienced APPs: learning approaches to common clinical scenarios and updates to improve AS in practice.

**Conclusions:**

Curricular content should acknowledge the cultural influences of the institution, target knowledge gaps and areas of interest of learners, and be delivered via flexible and engaging learning modalities that encourage maximal participation.

## Introduction

Antimicrobials are prescribed widely by providers in both subspecialty and general practice to treat infections. However, a significant proportion of antimicrobials prescribed are non-essential and contribute to resistance and adverse side effects. The CDC core elements of hospital antimicrobial stewardship programs (ASPs) were introduced in 2014 to improve prescribing, limit unnecessary use and patient harm, and combat resistance.^1^ Education is a core element of an ASP and should be tailored to the actions most relevant to the target provider group to have a meaningful impact on prescribing practices.

Education for antimicrobial prescribing varies in the medical community despite having a high rate of overuse. Most published antimicrobial stewardship (AS) education interventions have focused on medical students,^2–4^ medical residents, and infectious diseases (ID) fellows^5,6^ with few studies centered on advanced practice providers (APPs).^7,8^ APPs, including nurse practitioners (NPs) and physician assistants (PAs), represent a growing group of providers in pediatric inpatient and outpatient settings, and have varied backgrounds and educational experiences. Sym et al found that 66.3% of NP programs include only five hours or less of teaching on antimicrobials in a pharmacology course.^9^ Lee et al demonstrated similar findings with 53% of neurocritical care and cardiac surgery APPs at a single center reporting less than 10 hours of antimicrobial education in master’s level training programs.^7^ There is even less known about education on AS in APP programs. Similarly, there have been limited studies investigating APP knowledge gaps and interests regarding AS to guide the development of educational initiatives.^10,11^

Involving APP leadership in the development of education can allow educators to customize learning initiatives to fit the unique needs of the institution while also considering providers’ interests and varied backgrounds.^12^ While understanding the knowledge, attitudes and perceptions of APPs is vital to improving antimicrobial utilization long term, the perspective of key stakeholders of ASPs who help guide clinical decision-making is also necessary to identify target areas for educational interventions. Literature that considers the perspectives of APPs and ASP stakeholders is limited but necessary to optimize AS educational efforts. Our study aims to explore attitudes, prescribing practices, and knowledge deficits in AS, and barriers to learning among APPs through a qualitative needs assessment from the perspectives of APPs and ASP stakeholders to inform curriculum development at a large academic children’s hospital.

## Methods

### Setting

This single center qualitative study consisted of focus groups with APPs and ASP stakeholders at Children’s Hospital of Philadelphia (CHOP).

The ASP consists of three pediatric ID attending medical directors, three clinical pharmacists and several pediatric ID fellows. The ASP leads a prospective audit and feedback program where positive cultures and new antibiotic prescriptions are reviewed Monday through Friday, and recommendations are made directly to ordering providers. The ASP team participates in AS handshake rounds three times weekly in the general and subspecialty pediatrics units, pediatric intensive care unit (PICU), cardiac intensive care unit (CICU) and the cardiac care unit. Handshake rounds allow for in-person feedback and targeted education to be given to clinical teams caring for patients identified via prospective audit and feedback.^13^ The ASP also approves the use of restricted antimicrobials on a case-by-case basis from 7 AM–10 PM daily. Most antimicrobials require approval from the ASP to prescribe with exceptions for limited preapproved indications.

### Recruitment

Participants were recruited from October 9 to November 15, 2024. The project was discussed initially with APP clinical team leaders in the neonatal intensive care unit (NICU), PICU, CICU, and Division of Oncology to characterize the need for AS educational initiatives. APPs from these divisions were deliberately recruited given their higher usage of antimicrobials and frequent interactions with the ASP when prescribing restricted antimicrobials. Once a clear need for this education was identified, potentially eligible APPs were notified of the project in various ways: (1) APP clinical team leaders informed APPs in their divisions through word of mouth and electronic newsletters, (2) flyers with information for registration were posted in APP workrooms, (3) direct verbal communication from the study lead (N.H.) while conducting AS handshake rounds, and (4) individual informational emails to APPs with a registration link. Nine ASP key stakeholders were invited to participate via e-mail based on level of interaction with APPs through the AS pager by study lead (N.H.). This study was considered exempt by the CHOP Institutional Review Board.

### Focus groups

Focus groups were chosen for their ability to facilitate the exploration of perceptions, attitudes and feelings while allowing for clarification of views, reflection and rich group discussion.^14,15^ APP focus groups were hosted virtually to improve accessibility for maximal participation.

Four focus groups were comprised only of APPs, in which 11 open-ended questions were asked, including questions about education, training, influences on prescribing practices, knowledge gaps, and desired topics for education in AS, as well as general barriers to ongoing learning (Supplement 1). Questions were reviewed by a study team member (K.M.) and a NICU attending and piloted on a third-year internal medicine resident. Additional probing questions were iteratively added to the focus group guide as themes began to emerge through constant comparison for subsequent focus groups. One focus group included 1 ASP medical director, 3 ASP pharmacists and 2 first-year pediatric ID fellows hosted as a hybrid virtual/in-person meeting. The guide for this focus group included 5 open-ended questions about teaching initiatives, knowledge gaps, and high yield teaching topics for APPs from their perspective (Supplement 2).

Verbal consent was obtained from all participants prior to participation. Four to six participants were included in each focus group. Focus groups were facilitated by one study team member (N.H) who ensured all participants were given a chance to answer each question and moderated discussions. Focus groups continued until thematic saturation was achieved with no new themes or topics emerging during the discussion. Only one focus group was held with ASP stakeholders due to limited overlapping availability of participants. All participants in the study were given remuneration in the form of a $10 gift card for completion.

### Data collection

Focus groups were audio recorded via Microsoft Teams^TM^ and a voice recorder. Audio recordings of focus groups were transcribed by Microsoft Teams^TM^, validated and edited to ensure accuracy of transcription, and de-identified by one study member (N.H.).

### Analysis

Conventional content analysis with an inductive coding approach was used to develop the initial codebook after completion of all focus groups.^16^ Two focus group transcripts were double coded each by two team members (N.H. and J.R. or M.E.) using the Dedoose web application.^17^ Study team members discussed discrepancies with assigned codes, used axial coding to develop connections between codes, and refined the codebook.

An inter-rater reliability was calculated across 9 codes with a median κ of .78 (.76–1) and median percent agreement of 95%. The remaining transcripts were coded by one study member (N.H, J.R or M.E) and reviewed by another study member, with disagreements in applied codes discussed as a group.

## Results

**Figure 1.**
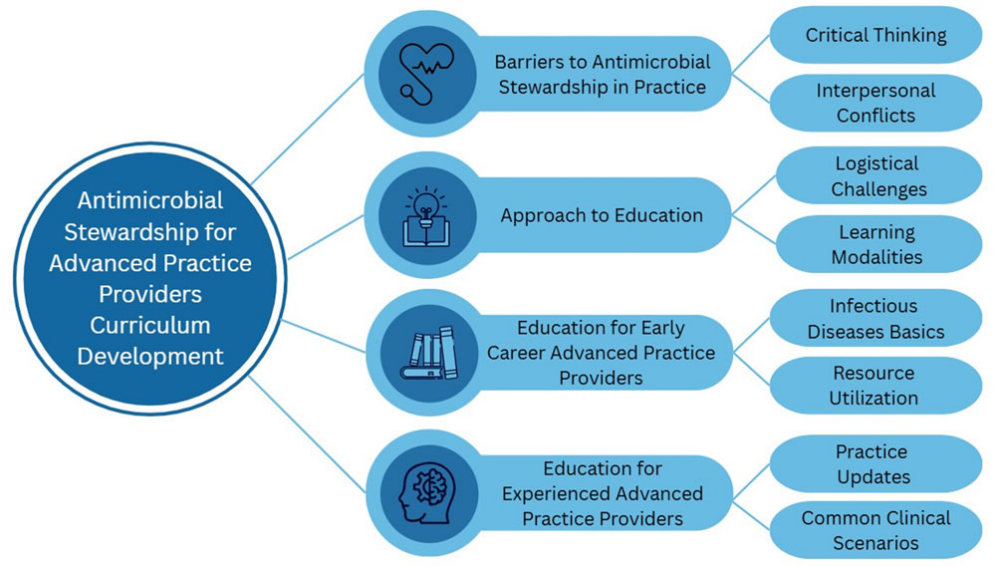
Domains and themes identified for antimicrobial stewardship curriculum development.

In total, 20 APPs participated in four focus groups (Table 1). 6 ASP key stakeholders participated in one focus group, including one medical director, two pediatric ID fellows and three pharmacists. Focus groups lasted 34 to 56 minutes (average time 47 mins). Four domains were generated with eight themes (Figure 1) with additional representative quotations included in Table 2.

**Table 1.** Demographic characteristics of advanced practice providers

Demographic characteristic	n (%)
**Position**	**n = 20 (%)**
Nurse practitioner	18 (69%)
Physician assistant	2 (8%)
**Division**	**n = 20 (%)**
Neonatal intensive care	5 (25%)
Pediatric intensive care	6 (30%)
Oncology	7 (35%)
Cardiac intensive care	2 (10%)
**Years in practice**	**n = 20 (%)**
0–5	5 (25%)
6–10	6 (30%)
11–15	1 (5%)
16+	8 (40%)
**Years at Children’s Hospital of Philadelphia**	**n = 20 (%)**
0–5	7 (35%)
6–10	6 (30%)
11–15	2 (10%)
16+	5 (25%)

**Table 2.** Representative quotations of domains and themes

Domains	Themes	Representative quotations
AS in practice	Interpersonal conflicts	“We have the surgeons who will just say ‘we want this many days of this antibiotic’ and there’s not really a rhyme or reason to it most of the time beyond the typical 24 hours of postoperative cefazolin or whatever. And so in those situations we’ll ask them why. And typically they say ‘because this is what I want’ and you just have to sort of make that work and then somehow get approval for it or not and have further conversations down the road.” APP12 “Sometimes there’s a lot of back and forth or they just say ‘thanks this is super helpful’ and sometimes ‘we just want what we want. Just stop asking all these questions’.” ASP1
	Critical thinking	“…understanding the ‘why’… I think it’s really important because if you just teach them this is what you use for CAP, then there’s a pattern recognition and not actually understanding of again like site, source as well as likely organisms there based on age groups.” APP7 “I think that the APPs should be open to asking us what we think when they’re not sure because a lot of times they will reach out for an approval request I end up asking them ‘why are you choosing this based on this culture? It looks like, you know, this might be the best choice’. And depending on the APP, they might be open to it, or they’ll tell me ‘that’s what my attending wants’. But I think they should be willing to ask us and have that discussion with us too, because I don’t have those types of discussions as often. It’s usually a ‘this is what I want’ request and I don’t see what their thought process is.” ASP4
Approach to education	Logistical challenges	“Trying to find time to fit things into our business schedules is challenging. So if we can have modes of education that can be flexible, like online seminars or on demand learning” APP9 “…doing it is a big undertaking, right? Figuring out where to start. Who are the people? Where do you get the APPs? There’s people in all different places and what is just even beginning to think through the logistics of who would we talk to? Who should we educate? When do they have time? Do they get continuing medical education credit to incentivize them? What does that look like for APPs? So all of that has just not been a priority because of competing priorities.” ASP2
	Learning modalities	“You could even do some sort of mixed modality. So a recorded lecture and then a live case study session or something may be really effective to minimize the time that people have to spend scheduling something in their day.” APP7
Education for early career APPs	Infectious diseases basics	“I think sometimes, especially for newer APPs, would be just the basics. I mean, you get some basics in school, but as we all know, sometimes you’re just treading water and you don’t take it in a lot of it. So, you know, just a good ‘these are your gram negatives. These are your gram positives. This covers this’.” APP1 “We probably don’t do a great job of explaining the importance of antibiotic resistance, antibiotic related adverse events, the microbiome, to the basics of why do we bother talking about this? I’ve had conversations, sometimes we say ‘well, you want to pick the narrowest antibiotic’ and they say ‘but why?’. So we need to take a few steps back. So I think even that as a starting point, I think is probably missing for a lot of new APPs.” ASP2
	Resource utilization	“I think also physically pointing people in the direction for a new APP of what resources are available. Like APP18 said, the pathways, and I know that that’s just one thing, but just knowing the resources is half the battle.” APP20 “I think the low hanging fruit is access to resources and navigating those, right? So finding pathways that have been worked on very hard and finding details on the AS program website even though it’s behind multiple layers. But their means to get to that would be, I think in their early education.” ASP5
Education for experienced APPs	Practice updates	“I think for new people the updates may not be as important as they’re fresh out of school, but I’m two years out of school and I already feel like I need to brush up… the recommendations change all the time. And once you’re out of school, other than subscribing to these journals, there really isn’t a great way for you to get that information.” APP17
	Common clinical scenarios	“Something case based is very practical. And it could include not just how you make your antibiotic choices, but how we get this information. We go look at this resource, this is how we got there. And then we could go back and we do this. And then we reached out to our infectious diseases colleagues and they asked us these questions. And I think that there there’s an opportunity to really broaden the learning, not just ‘this is the bug and this is the bug juice that you get to treat it.’” APP11 “I think it depends on what area they’re working in and what they’re seeing most often. The intensive care unit APPs probably need to know more about like how to diagnose CAP, VAP, CLABSI, and sepsis not otherwise specified, and how to take it from sepsis not otherwise specified to actually diagnosing a thing? Whereas the urgent care, primary care and emergency department it’s probably more like otitis, sinusitis, how to differentiate viral respiratory tract infection from something that needs antibiotics. Everybody needs to learn UTI.” ASP2

AS, antimicrobial stewardship; ASP, antimicrobial stewardship program; APP, advanced practice provider; CAP, community acquired pneumonia; VAP, ventilator associated pneumonia; CLABSI, central line associated bloodstream infection; UTI = urinary tract infection.

### Barriers to AS in practice

Several barriers to implementing AS into clinical practice were emphasized by participants. Two themes were identified, including interpersonal conflicts and critical thinking.

APPs frequently recounted being given instructions regarding use of antibiotics by their primary attending or a consulting team where they did not understand and/or agree with the recommendations. They highlighted tension with the ASP when requesting approval for use of antibiotics in these situations and described being “between a rock and a hard place” (APP11, APP13) or “the middle man” (APP12, APP17, ASP1). Similarly, ASP stakeholders recalled interactions with APPs requesting approval for antimicrobials that they felt were inappropriate and sensed a lack of openness from APPs to discussing alternatives. APPs report there is no education on how to navigate these types of disagreements despite frequent situations where APPs, the ASP, and consulting teams are “fighting over antibiotics” (APP16). Additionally, APPs also felt that having restrictions in place for prescribing antimicrobials hindered efficiency, and in some cases the depth to which they considered treatment options for their patients. One participant stated, “if I’m lazy I’ll just take whatever infectious diseases tells me to do. But it would be nice to grow and learn” (APP6).

APPs described in detail the various patient, microbiological, and medication factors they consider and how they incorporate them into patient care decisions. However, many APPs expressed discomfort when caring for patients whose clinical presentation did not fall under a standard operating procedure or pathway. Similarly, they noted that their less experienced APP colleagues inappropriately relied largely on pattern recognition for clinical decision-making, stating, “I think we have to learn to think beyond just pathways” (APP7). ASP stakeholders noted that there are significant knowledge gaps among APPs in consideration of a patient’s clinical context, and ordering and interpretation of diagnostic testing that AS practices. As one participant commented, “interpretation of diagnostics is also a problem. They say, ‘this test is positive, it needs treatment’. And that’s not really how that works” (ASP2).

### Approach to education

Logistical challenges and preferred learning strategies for effective education were identified by APPs. The most frequently cited barrier was lack of time. Specifically, APPs expressed that their schedules have limited flexibility, making in-person lessons difficult to arrange for larger groups. They also reported numerous interruptions related to patient care during education that took place during regular work hours. APPs expressed that offering options for distance learning, including participating virtually in real time, or having access to recorded lessons, could mitigate this challenge. Pros and cons of preferred learning formats were discussed. There was widespread dissatisfaction with module-based learning, which is a dominant form of APP Continuing Medical Education (CME). When discussing module-based education, one participant said, “I just want to click through, complete it and move on because I’m busy” (APP10). Many participants favored education that is case-based given its promotion of rich discussion among small and large groups that resulted in both deeper understanding and lasting change.

Participants also described the importance of being taught topics related to their specialty, interests, and level of education as crucial for engagement. Incentives to engage meaningfully with education were also highlighted. Many APPs expressed that education qualifying for CME credit, specifically in pharmacotherapeutics for NPs, is a significant incentive for participation.

The ASP stakeholders highlighted several reasons why a formal curriculum has not been developed for APPs thus far. Lack of time and knowledge of how to develop and implement such a curriculum were discussed as the main barriers. ASP stakeholders also felt unsure of the educational background and interests of APPs, noting that APP training is “obviously very different from Doctor of Medicine/Doctor of Osteopathic Medicine training and Doctor of Pharmacy training and it definitely varies by APP” (ASP2).

### Education for early career APPs

Participants highlighted that the learning needs of early career APPs are different from those of experienced APPs. Specific topics identified are outlined in Table 3. Notably, difficulty with identifying and utilizing resources that are widely available and specific to CHOP for treatment of infections and AS was described by many participants. The CHOP ASP intranet web page contains the antibiogram, institution specific guidelines, diagnosis-specific pathways, and AS decision-making tools, but was described by APPs as “overwhelming” (APP12, APP13) and “not very satisfying” (APP11). Similar concerns were raised by APPs about lack of knowledge regarding which general resources were most appropriate to use to aid in decision-making. The ASP team emphasized a similar need for APPs to learn how to use published resources and highlighted the underuse of the ASP team as a resource. ASP stakeholders expressed that they “want APPs to know who we are when they are onboarding…we are here to help them with any antibiotic questions” (ASP4).

**Table 3.** Major topics of interest by level of experience for advanced practice providers

Early career advanced practice providers	Experienced advanced practice providers
**Infectious diseases basics** 1. Characteristics of g and g bacteria 2. Bacterial colonization and the human microbiome 3. Mechanisms of infection 4. Antibiotic spectrum of activity in clinical practice 5. Syndrome specific diagnosis and management (e.g.,: acute otitis media, sinusitis, urinary tract infections, pneumonia)	**Common clinical scenarios** 1. Antimicrobial Stewardship Skills: interpreting the antibiogram and culture results, recognizing resistance patterns 2. Diagnostic Stewardship Challenges: culture negative sepsis work-up, respiratory cultures, urine cultures 3. Challenging Cases in Infectious Diseases: when patients don’t fit on a clinical pathway
**Resource utilization** 1. Foundational Principles of Antimicrobial Stewardship 2. Navigating institution specific antimicrobial stewardship resources: antibiograms, clinical pathways 3. Navigating general antimicrobial stewardship resources (e.g.,: American Academy of Pediatrics Red Book, Lexicomp) 4. Communicating effectively with consulting teams and antimicrobial stewardship	**Practice updates** 1. Major practice changes in diagnosis and management of infections by specialty (e.g.,: neonatal intensive care, general pediatrics) 2. Institution specific initiatives in antimicrobial stewardship

Both APPs and ASP stakeholders expressed a need for early career APPs to learn basic principles in the clinical application of microbiology, antimicrobials, and ID before learning about AS practices. ASP stakeholders noted that teaching AS principles to new-to-practice APPs is difficult because “there’s a lot of missing general infectious diseases knowledge, which makes it hard to teach stewardship” (ASP2). APPs also reported significant variation in their early training when learning about microbiology, antimicrobials, and ID. One APP recalled feeling “really well prepared…it sounds like there’s not much that has changed about antibiotics over the last 20 years” (APP19). Another APP reported their formal training “minimally prepared me…I’m not comfortable with these decisions” (APP16). Several APPs reported their training in antimicrobials was limited within their pharmacology classes, and most reported no dedicated education on AS. APPs specifically expressed a desire to learn about characteristics of g and negative bacteria, and antimicrobial spectrum of activity for treatment. One participant described struggling with “when you have a bug that’s susceptible to multiple antibiotics, figuring out what is more narrow, especially for the cephalosporins because they all look the same” (APP9). Focusing on syndrome-based teaching, particularly for commonly encountered, uncomplicated conditions was highlighted by participants as important earlier in APP education.

### Education for experienced APPs

APP participants emphasized the need to learn several AS skills and expressed a desire to incorporate them into practice, including how to use antibiograms, interpret culture results, recognize resistance patterns, narrow antibiotics for definitive therapy and select durations of treatment. Specific topics of interest are listed in Table 3. One participant stated, “if you’re susceptible to several antibiotics, understanding why did you choose antibiotic A or B or C” (APP6) as important to understand. Similarly, ASP6 noted, “being able to interpret cultures would be a very useful skill to reinforce…at least general overviews of what type of susceptibility profiles we might expect for certain organisms, what should we be on the lookout for, and when a susceptibility pattern indicates having to broaden.”

Participants discussed many desired education topics for experienced APPs relating to general and specialty-specific practice. Exploring the nuances of clinical decision-making using case examples was highlighted as valuable for understanding the ID team perspective, and “guiding the critical thinking” for complex patients (APP6). ASP stakeholders endorsed a need for APPs to conceptualize patients’ clinical syndromes and consider “what am I actually worried about? Which is the right test for that…If you don’t have the syndrome right, you’re never going to be able to get the right antibiotic either” (ASP2). Specific clinical scenarios were brought up by APPs and ASP stakeholders as particularly important, including culture negative sepsis, pneumonia, sinusitis, acute otitis media, and urinary tract infections. All participants agreed that targeting cases to the audience and their specialty of practice would be most engaging.

Several participants expressed a desire for education to help keep their practice up to date. Many APPs expressed difficulty with learning about advances in CHOP-specific practices, antimicrobial treatment options, durations of therapy, emerging pathogens, resistance patterns, and geographic updates in the local region.

## Discussion

This study is the first of its kind to assess education needs from the perspectives of both APPs and ASP stakeholders and makes important contributions to the consideration of APP education in AS. First, we identified numerous knowledge gaps and delineated important topics for education based on APP level of experience and scope of practice. This allows for the creation of multiple novel curricula that are learner centered. There was overall agreement among APP and ASP stakeholders that emphasis should initially be placed on development of curriculum specific to new-to-practice APPs. Prior survey-based assessments of APPs and AS demonstrated general education needs, particularly in using antibiograms, understanding antibiotics, de-escalating therapy, and identifying resistant organisms.^10,11,18^ Needs assessments of pediatric physicians identified a wide range of important topics for education including understanding antimicrobial spectrum of activity and local antibiotic resistance patterns, selecting empiric antibiotic, using clinical decision support tools, therapeutic drug monitoring, interpreting culture results, and determining durations of therapy.^18–20^ Broader assessments of a range of providers have also demonstrated a need for education on diagnostic stewardship.^21^ While several of these topics and more were also identified as knowledge gaps for APPs in our study, we additionally clarified the educational background and practice environment of APPs, as well as ASP stakeholders’ understanding of APP practice, providing valuable context for creating educational materials that meet the unique needs of this group of learners.

Second, we identified key elements of the social learning environment as they relate to AS that must also be addressed in curricula development. Social interactions, the community, and desired behaviors of the institution that are often invisible provide valuable context to individual prescriber decisions regarding antimicrobials and must be addressed to promote meaningful changes in prescribing practices.^22–24^ This “prescribing etiquette” of unwritten yet widely accepted cultural rules regarding antimicrobials is strongly influenced by social norms that emerge from unit-based and organizational subcultures.^25^ Pressure from attendings to prescribe antibiotics that a provider does not think are necessary is prevalent, particularly among junior staff.^26^ Senior physician preferences, expectations and prescribing norms also shape junior doctors’ prescribing decisions and they risk facing social sanctions if they make decisions that do not align with these norms.^25,27^ Interestingly, there is a paucity of literature discussing interpersonal issues between primary providers, including APPs, and multiple subspecialty teams. Participants of this study highlighted this as having a significant influence on prescribing habits and a source of tension in relationships with members of consulting teams. This is an important area of future investigation to thoroughly explore the cultural influences of the institution as it relates to provider identity, autonomy, education, and clinical decision-making.

Interpersonal relationships and communication between providers and members of the ASP is also a complicated issue that can hinder AS in practice. The ASP approval process for restricted antimicrobials has been found to decrease efficiency.^18,23,28^ Many providers engage in workarounds, particularly to satisfy demands of their attending or specialty consult service when there are disagreements, which is a phenomenon that was frequently reported by APPs and ASP stakeholders in this study.^18,25,28^ There are conflicting reports on how restricting antimicrobials and AS handshake rounds impact the relationship between providers and the ASP. Some studies report these interventions have bred antagonism between the ASP and providers, invoked threats to autonomous prescribing, and undermined teamwork, while others had opposite results.^18,25,29^ This phenomenon was discussed among participants, and interestingly, some APPs also noted that these measures, particularly the requirement for preapproval to prescribe antimicrobials, led them to defer critical thinking about antimicrobial choices to the ASP.

Third, we identified incentives, barriers and facilitators for learning for APPs with diverse experiences. There is significant variability in teaching resources that APPs find most useful for ID and AS education. Live/virtual recorded lectures (93%) and medical journals (85%) have been rated as most useful for continuing education on antibiotics, with ASPs as a resource ranked slightly lower (77%) by NPs.^11^ Survey-based studies showed lower utilization of the ASP as a source for education.^10,30^ In contrast, APPs participating in a pilot AS education program cited lectures as the least utilized source for education and relied primarily on peer-reviewed point of care resources.^7^ Participants of this study expressed a need to educate APPs on the utility of the ASP as a resource for clinical decision-making. This lack of awareness could be due to many factors including low exposure to the ASP on certain clinical units and poor explanation of the role of the ASP as a resource for AS practices. The variability in resources for learning may exist because AS education is typically targeted toward medical students and residents with different educational backgrounds and roles.^12^ Interestingly, despite the rise in popularity of online asynchronous education for time-limited learners, participants in this study unanimously expressed dissatisfaction with online module-based learning in overall engagement and retention. Teaching strategies that incorporate desired elements for learning could include role-playing, guided discussions, gamification, simulated clinical scenarios and concept mapping. Given time constraints highlighted by all participants, other modalities that rely on participation outside of teaching time such as problem based learning and flipped classroom may be less feasible.

This study is not without limitations. First, this needs assessment was conducted in a single center and thus may have unique findings relevant to the specific context of our hospital. This study also included APPs from specific divisions with higher rates of antibiotic use and thus the results may be less generalizable to APPs in all clinical settings. Additionally, most APP participants had more than five years of experience in practice and as such, results may favor those with more experience. However, many comments did consider the perspective and needs of new-to-practice APPs.

Overall, this needs assessment identified several important topics for a comprehensive AS curriculum for APPs with a focus on area of practice and level of experience. Both APPs and ASP stakeholders recognized that further education on AS is important for diagnosing and treating infections and educational content that considers the culture of the institution is essential for enabling true behavior changes. Next steps include developing an engaging curriculum to meet these described local needs utilizing recommended modalities of delivery. Given similar educational needs described in the literature, this curriculum can also be disseminated widely, impacting prescribing practices and improving antibiotic use far beyond the walls of our own institution.

## Supporting information

10.1017/ash.2025.10278.sm001 Hill et al. supplementary material 1 Hill et al. supplementary material

10.1017/ash.2025.10278.sm002 Hill et al. supplementary material 2 Hill et al. supplementary material

## Supplementary material

The supplementary material for this article can be found at https://doi.org/10.1017/ash.2025.10278.
