# Supplementary material for: Identified needs in antimicrobial stewardship education for pediatric advanced practice providers: a qualitative analysis
Source: Antimicrob Steward Healthc Epidemiol. 2026 Jan 20;6(1):e25. doi: 10.1017/ash.2025.10278 (PMC12835947; doi:10.1017/ash.2025.10278)
Supplement: Hill et al. supplementary material 1 — Hill et al. supplementary material [file S2732494X25102787sup001.docx]

**APP Focus Group Guide**

**Q1**. Tell me which division you work in, how many years you have been in practice, and how many of those years have been at CHOP

**Q2**. What education/training have you had in antimicrobial stewardship in NP/PA school and since entering practice?

- Probe: tell me about antimicrobial stewardship education you received while working at other institutions and at CHOP

**Q3**. How did the training and education you received prepare you for practice?

**Q4**. What patient factors do you consider when prescribing an antibiotic?

- Probe: Which factors are most important for picking an antibiotic for a patient?

**Q5**. What team-based factors can influence antibiotic selections?

- Probe: how do your team’s opinions influence your decision making?
- Probe: how do other clinical teams’ opinions influence your decision making?

**Q6**. What resources do you use to guide antibiotic selection?

- Probe: how do you use the following resources:
  - residents, attendings, fellows, pharmacists
  - antimicrobial stewardship group
  - CHOP Antibiogram or clinical pathways.
    - How do you feel when a patient’s presentation does not align with a clinical pathway?
  - AAP Redbook, up to date, or other published resources?

**Q7**. Tell me about a time that you or your team made decisions about antibiotics where you did not understand the rationale.

- Probe: Are there clinical scenarios or syndromes that come up where you feel like ID or another consulting team’s decision making did not make sense or agree with your plan?

**Q8**. What topics would you like to see taught in antibiotic stewardship education curriculum?

- Probe: would what do you think about the following as learning topics:
  - how to use resources (e.g. pathways, antibiogram)?
  - how to interpret culture results?
  - antibiotic side effects and dosing?
  - de-escalate antibiotics?
  - IV and oral drug conversions?

**Q9**. Which topics are most important to cover in your opinion?

- Probe: what should be prioritized for a new APP vs an experienced APP?

**Q10**. What do you identify as the most significant barriers to your learning?

- Probe: is there a way that you think delivering curriculum works best to reach as many people as possible and is user friendly?

**Q11**. Do you have any thoughts or remarks that you wanted to mention that you felt were important that I didn’t ask about in terms of antimicrobial stewardship and education?
