# Supplementary material for: Identified needs in antimicrobial stewardship education for pediatric advanced practice providers: a qualitative analysis
Source: Antimicrob Steward Healthc Epidemiol. 2026 Jan 20;6(1):e25. doi: 10.1017/ash.2025.10278 (PMC12835947; doi:10.1017/ash.2025.10278)
Supplement: Hill et al. supplementary material 2 — Hill et al. supplementary material [file S2732494X25102787sup002.docx]

**ASP Stakeholder Focus Group Guide**

**Q1**. Tell me about any teaching initiatives that you've done, both formal or informal, that have been geared towards APPs at CHOP.

- Probe: What is your impression of the effectiveness of the teaching you have done?

**Q2**. What are some of the barriers you have encountered with implementing antimicrobial stewardship teaching for learners?

- Probe: What is your impression of our relationship as the antimicrobial stewardship team with APPs and openness and willingness to learn?

**Q3.** What knowledge gaps have you identified in APPs?

- Probe: have you identified common questions that come to ASP on EPIC/Haiku? How about consult questions or on handshake rounds?

**Q4**. What do you feel are important topics in antimicrobial stewardship that should be taught to APPs?’

- Probe: which education should be targeted towards new APPs versus experienced APPs?
- Probe: which clinical syndromes would you say are ones that need to be taught first?
- Probe: what do you think about teaching resource utilization to APPs?
- Probe: do you think teaching about antimicrobial IV to PO conversions, side effects, dosing and durations of therapy could be helpful?
- Probe: do you think teaching how to interpret culture reports and use the antibiogram could be helpful?

**Q5**. Is there anything that anybody wanted to bring up that they thought was important on that topic that I didn't ask you about or you didn't get a chance to say?
